# Neuronal haemoglobin induces loss of dopaminergic neurons in mouse Substantia nigra, cognitive deficits and cleavage of endogenous α-synuclein

**DOI:** 10.1038/s41419-022-05489-y

**Published:** 2022-12-16

**Authors:** Chiara Santulli, Carlotta Bon, Elena De Cecco, Marta Codrich, Joanna Narkiewicz, Pietro Parisse, Fabio Perissinotto, Claudio Santoro, Francesca Persichetti, Giuseppe Legname, Stefano Espinoza, Stefano Gustincich

**Affiliations:** 1 Area of Neuroscience, Scuola Internazionale Superiore di Studi Avanzati (SISSA), Trieste, Italy; 2 Central RNA Laboratory, Istituto Italiano di Tecnologia (IIT), Genova, Italy; 3 Elettra – Sincrotrone Trieste S.C.p.A., Trieste, Italy; 4 Istituto Officina dei Materiali – Consiglio Nazionale delle Ricerche, Trieste, Italy; 5 Department of Health Sciences and Research Center on Autoimmune and Allergic Diseases (CAAD), University of Piemonte Orientale (UPO), Novara, Italy

**Keywords:** Molecular neuroscience, Parkinson's disease

## Abstract

Parkinson’s disease (PD) presents the selective loss of A9 dopaminergic (DA) neurons of Substantia Nigra *pars compacta* (SNpc) and the presence of intracellular aggregates called Lewy bodies. α-synuclein (α-syn) species truncated at the carboxy-terminal (C-terminal) accumulate in pathological inclusions and promote α-syn aggregation and toxicity. Haemoglobin (Hb) is the major oxygen carrier protein in erythrocytes. In addition, Hb is expressed in A9 DA neurons where it influences mitochondrial activity. Hb overexpression increases cells’ vulnerability in a neurochemical model of PD in vitro and forms cytoplasmic and nucleolar aggregates upon short-term overexpression in mouse SNpc. In this study, α and β-globin chains were co-expressed in DA cells of SNpc in vivo upon stereotaxic injections of an Adeno-Associated Virus isotype 9 (AAV9) and in DA iMN9D cells in vitro. Long-term Hb over-expression in SNpc induced the loss of about 50% of DA neurons, mild motor impairments, and deficits in recognition and spatial working memory. Hb triggered the formation of endogenous α-syn C-terminal truncated species. Similar α-syn fragments were found in vitro in DA iMN9D cells over-expressing α and β- globins when treated with pre-formed α-syn fibrils. Our study positions Hb as a relevant player in PD pathogenesis for its ability to trigger DA cells’ loss in vivo and the formation of C-terminal α-syn fragments.

## Background

Parkinson’s disease (PD) is a chronic progressive neurodegenerative disorder clinically defined in terms of motor symptoms. The most evident pathological hallmarks are the selective degeneration of A9 dopaminergic (DA) neurons of Substantia Nigra *pars compacta* (SNpc) and the presence of intracellular aggregates called Lewy bodies (LB). The consequent loss of DA synapses in the striatum is the primary origin of the inability to control movements [1]. The causes of the selective degeneration of A9 neurons remain largely unknown.

Highly penetrant mutations producing rare, monogenic forms of the disease are in genes involved in mitochondria homoeostasis, vesicular trafficking and lysosomal function [2]. The discovery of missense mutations or duplication/triplication of the α-synuclein gene (SCNA) proves its causative role in autosomal-dominant early-onset PD [3]. α-synuclein (α-syn) protein is the major constituent of LB with a mix of the full-length protein (FL-α-syn) and C-terminal truncated fragments (ΔC-α-syn). Progressive α-syn aggregation can be recapitulated in model systems by the exogenous introduction of in vitro generated preformed fibrils (PFFs) [4–6]. PFFs seed the aggregation of endogenous α-syn with a prion-like mechanism and this aggregation may spread along synaptically connected pathways [4, 7–9].

In the quest of the molecular basis of neurodegeneration, the vulnerability of DA cells of the SNpc was associated to genes differentially expressed in comparison to DA neurons of the Ventral Tegmental Area (VTA; A10), largely spared in disease. Transcripts and encoded proteins for α-and β-chains of Haemoglobin (Hb) are enriched in A9 DA neurons of mouse SNpc [10–12]. This pattern of expression is conserved in the human mesencephalic DA cells’ system [13]. Neuronal Hb (nHb) retains its α_2_β_2_ tetrameric structure as in blood where it exerts its function as an oxygen-carrier molecule [12]. In the human brain, α-and β-chains are co-localized in the mitochondrion and interact with mitochondrial proteins [14]. Mitochondrial Hb is significantly lower in aged monkey striatum and human SN [15, 16] as well as in PD post-mortem brains [16, 17]. This decrease is caused by the formation of Hb/α-syn complexes reducing the pool of free nHb.

The co-overexpression of α-and β-chains in the mouse DA iMND9 cell line in vitro and in mouse SNpc by AAV-mediated delivery in vivo has proved that Hb may interfere with cellular pathways targeted in PD [11]. In vitro, Hb overexpression alters transcript levels of genes involved in oxygen (O_2_) homoeostasis and oxidative phosphorylation suggesting a role in mitochondrial activity [11]. It also increases DA cells’ susceptibility to 1-methyl-4-phenylpyridinium (MPP+) and rotenone [11], neurochemical in vitro models of PD targeting mitochondrial complex I activity [18]. Upon stress, Hb inhibits autophagy, and it moves to the nucleus where it forms insoluble aggregates in the nucleolus triggering nucleolar stress [11].

When AAV carrying α- and β-chains of Hb are stereotaxically injected into mouse SN, Hb forms cytoplasmic and nucleolar aggregates, one month after injection [11]. While no effects on DA neurons number are evident, a motor learning impairment is observed, urging the need for further analysis at longer time points.

Here, we show that a prolonged ectopic expression of Hb in rodent SNpc induces the loss of DA neurons along with motor and cognitive impairments. Linking Hb to molecular pathways involved in PD, Hb overexpression in vivo and in vitro provokes the formation of ΔC-α-syn, a post-translational modification (PTM) associated with PD. These results provide new evidence in support of the role of Hb in PD pathogenesis.

## Materials and methods

### Animals

All animal experiments were performed in accordance with European guidelines for animal care and following Italian Board Health permissions (D.Lgs. 26/2014, 4 March 2014). Mice were housed and bred in IIT – Istituto Italiano della Tecnologia (Genova, GE, Italy) animal facility, with 12 h dark/night cycles and controlled temperature and humidity. Food and water were provided *ad libitum*.

### Behavioural testing

All procedures involving animals and their care were carried out in accordance with the guidelines established by the European Community Council (Directive 2010/63/EU of September 22, 2010) and were approved by the Italian Ministry of Health (DL 116/92; DL 111/94-B). Sample sizes used in the present study were based on previous behavioural studies that showed the minimum number of mice needed to show a significant difference. Investigators were blind to mouse treatment group during behavioural testing.

### Locomotor activity

To measure spontaneous locomotor activity, mice (CTRL *n* = 12; Hb *n* = 12) were placed in the locomotor activity chambers (Omnitech Digiscan, Accuscan Instruments, Columbus, ОН, USA) for 60 min and the total distance travelled was measured by the analysis of infra-red beam interruptions.

### Rotarod

A Rotarod from TSE Systems was used. Briefly, mice (CTRL *n* = 15; Hb *n* = 15) were handled on alternate days during the week preceding the start of the Rotarod test (3 handling sessions; 1 min per mouse per session). Behavioural testing lasted two days. On day 1 mice habituation to rotation on the rod under a constant speed of 4 r.p.m. for three trials (60-s inter-trial interval) was performed in the morning. The trial ended as the mouse was falling off the rod or if it was spending on the rod more than 300 s. In the afternoon, mice were placed on the rod having a constant 4 r.p.m.-speed for 60 s. Then, the accelerating programme was launched for three trials (60-s inter-trial interval). The trial ended as previously described. Mice were tested the day after only as for day 1 afternoon. Time spent on the rod was automatically recorded. The average time spent on the rod was then calculated.

### Static rods

Five 60 cm long wooden rods of varying thicknesses (35, 25, 15, 10, and 8 mm diameter) were fixed to a laboratory shelf horizontally protruding into space at 60 cm of height above the floor. Mice (CTRL *n* = 15; Hb *n* = 15) were placed outward at the far end of the widest rod. Two measures were considered: orientation time (time taken to orientate 180° from the starting position towards the shelf) and transit time (the time taken to travel to the shelf end). Orientation was dependent on the mouse staying upright. The maximum score of 120 s was assigned when the mouse turned upside down and clung below the rod, fell or reached the maximum test time. If the mouse fell off the rod within 5 s, another attempt was allowed (as falling within 5 s could be due to faulty placing by the experimenter), for a maximum of three trials, and the best result was considered. Mice were not tested on smaller rods. Once tested on one rod, mice were placed back to the home cage to rest, while other mice were tested. This procedure was repeated for all the rods. The time for orientation and transit were plotted in the graphs for statistical analysis. Since in smaller rods many of the mice fell or did not complete the test, the success rate of the test was also calculated as the number of mice that completed the test and the Chi-square test was used to compare the two groups of mice.

### Horizontal bars

For this test, two bars made of brass were used, 40 cm long, held 50 cm above the bench surface by a wooden support column at each end. Two bar diameters were used: 2 and 4 mm. As mice find it easiest to grasp the narrow 2 mm bar, mice (CTRL *n* = 15; Hb *n* = 15) were first tested on this bar. Holding it by the tail, the mouse was placed on the bench in front of the apparatus, pulled quickly backwards about 20 cm (perpendicular alignment to the bar), rapidly raised and allowed to grasp the horizontal bar at the central point with its forepaws only and then the tail was released. The time to reach one of the edges of the bar was recorded. If the mouse had failed to grasp the bar properly at the first attempt or fell within 5 s, the score was not recorded, and it was placed back to the cage to rest and then the trial was repeated up to three times since a poor placement of the operator might occur. The best score was excluded from these additional trials. The score was calculated as an average of the scores of the 2 mm and 4 mm bar trials as indicated below:

Falling between 1 and 5 s = 1

Falling between 6 and 10 s = 2

Falling between 11 and 20 s = 3

Falling between 21 and 30 s = 4

Falling after 30 s = 5

The maximum score for completing the test was 5 for each bar and 10 for both bars.

### Novel object recognition test

Mice (CTRL *n* = 8; Hb *n* = 8) were handled on alternate days during the week preceding the start of the test (3 handling sessions; 1 min per mouse per session on day 1, 3, 5.). On day 6, mice were subjected to the habituation session in the empty open field for 1 h. The intensity of the light on the apparatus was about 60 lux. On day 7, each mouse was subjected to two successive sessions. Pre-test session (acquisition trial, 10 min): each mouse was introduced into the open field containing two identical copies of the same object. At the end of the session, the mouse was placed into the home cage. Test session (retention trial, 5 min): 1 h after the acquisition trial, both objects were substituted with a third copy of the previous object and a new object. The animals were considered to be exploring the object when they were facing (at a distance < 1 cm) or were touching or sniffing the object. Mice that explored object for < 4 s were excluded. The type and positions of presentation of the objects during the acquisition and retention phase were counterbalanced across animals. The preference index expressed as the ratio of the amount of time spent exploring the objects (training session) or the novel one (retention session) over the total time spent exploring both objects, defined the recognition memory. In the pre-test session, the total amount of exploration (in seconds) for the identical objects was used to verify no preference for one of the two side of the chamber where the objects were located.

### Y-maze spontaneous alternation test

The apparatus was a Y-shaped maze with three opaque arms spaced 120° apart with a measure of 40 × 8 × 15 cm each. An overhead camera was mounted to the ceiling directly above the apparatus to monitor mice movement and 4 standing lamps with white light bulbs were placed at the corners outside privacy blinds pointed away from the apparatus. The arms were labelled as A, B or C to identify the entries. The animal (CTRL n = 8; Hb *n* = 12) was placed inside arm B facing away from the centre and allowed to move through the apparatus for 10 min while being monitored by an automated tracking system. The trial began immediately and ended when the defined duration had elapsed. Scoring consisted of recording each arm entry (defined as all four paws entering the arm). The total entries in all arms were recorded. A spontaneous alternation occurred when a mouse entered a different arm of the maze in each of 3 consecutive arm entries. The % of spontaneous alternation was then calculated as ((#spontaneous alternation/(total number of arm entries-2)) × 100.

### Stereotaxic AAV9 injection

Adult (12 weeks old) male C57BI/6 J mice were used for experiments (CTRL *n* = 15; Hb *n* = 15). Mice were anaesthetized by a mixture of Isoflurane/Oxygen and placed on a stereotaxic apparatus (David Kopf instrument, Tujunga, CA, USA) with a mouse adaptor and lateral ear bars. The skin on the skull was cut and one hole was made on both sides by a surgical drill. A stereotaxic injection of 1 µL of viral vector suspension (AAV9-CTRL or a mixture of AAV9-2 x FLAG-α-globin and AAV9-β-globin-MYC, called AAV9-Hb; titre: 5 × 10^12^ vg/mL) was delivered bilaterally to SNpc at the following coordinates: anterior/posterior (A/P) −3.2 mm from bregma, mediolateral (M/L) −1.2 mm from bregma and dorsoventral (D/V) −4.5 mm from the dura. The coordinates were calculated according to the Franklin and Paxinnos Stereotaxic Mouse Atlas. The injection rate was 1 µL/15 min using a glass gauge needle. After the infusion, the needle was maintained for another 1 min in the same position and then retracted slowly.

### Tissue collection and processing

At 10 months after injection of AAVs into SNpc, the animals were sacrificed. Following induction of deep anaesthesia with an overdose of a mixture of Xylazine and Zoletil, the animals were intensively perfused transcardially with PBS 1×. For biochemical analysis, SNpc was dissected and immediately frozen in liquid nitrogen and stored at −80 °C, pending analyses. For immunohistochemical analysis, after the intensively transcardially perfusion with PBS 1×, animals were perfused with 4% paraformaldehyde diluted in PBS 1×. Brains were postfixed in 4% paraformaldehyde for 1 h at 4 °C. The regions containing the SN were cut in 40 μm free-floating slides with a vibratome (Vibratome Series 1000 Sectioning System, Technical Products International, St. Louis, MO, USA). Four consecutive series were collected to represent the whole area of interest.

### Immunofluorescence with labelled α-syn fibrils

Human α-syn fibrils were labelled with Alexa-488 succinimidyl ester (Thermo Fisher Scientific, A20000) following the manufacturer’s instructions and the unbound fluorophore was removed with multiple dialysis steps in sterile PBS 1×. Uptake experiments were performed following standard IF protocol or following the protocol described by Karpowicz et al [19]. Briefly, cells seeded on coverslips were incubated with the culture medium containing labelled α-syn fibrils for 24 h. Prior to fixation, fluorescence from non-internalized fibrils was quenched by incubating with Trypan Blue for 5 min. Cells were then fixed in 4% paraformaldehyde for 20 min, washed two times and permeabilized with 0.1% Triton X-100 in PBS 1× for 4 min and incubated with HCS Blue Cell Mask 1:1000 for 30 min (Thermo Fisher Scientific). Cells were washed twice in PBS 1× and once in Milli-Q water and mounted with Vectashield mounting medium (Vector Lab, H-1000). Images acquisition was performed using C1 Nikon confocal microscope (60× oil, NA 1.49, 7× zoom-in) as z-stacks of 0.5 µm.

### Immunocytochemistry

Cells were washed twice with PBS 1×, fixed in 4% paraformaldehyde for 20 min, washed twice with PBS 1× and treated with 0.1 M glycine for 4 min in PBS 1×, washed twice and permeabilized with 0.1% Triton X-100 in PBS 1× for 4 min. Cells were then incubated in blocking solution (0.2% BSA, 1% NGS, 0.1% Triton X-100 in PBS 1×), followed by incubation with primary antibodies diluted in blocking solution for 2:30 h at room temperature. After two washes in PBS 1×, cells were incubated with labelled secondary antibodies and 1 μg/ml DAPI (for nuclear staining) for 60 min. Cells were washed twice in PBS 1× and once in Milli-Q water and mounted with Vectashield mounting medium (Vector Lab, H-1000). The following antibodies were used: anti-FLAG 1:100 (Sigma–Aldrich, F7425), anti-MYC 1:250 (Cell Signaling, 2276), anti-α-syn (C-20) 1:200 (Santa Cruz Biotechnology, sc-7011-R), anti-α-syn (SYN-1) 1:200 (BD Transduction Laboratories, 610787) and anti-α-syn(phosphoS129) 1:200 (Abcam, ab59264). For detection, Alexa Fluor-488, −594 or −647 (Life Technologies) antibodies were used. Image acquisition was performed using C1 Nikon confocal microscope (60x oil, NA 1.49, 7× zoom-in). In the present study, data were collected from 6 independent experiments.

### Immunohistochemistry

For immunohistochemistry, free-floating slides were rinsed three times in PBS 1×, contained 0.1% Triton X-100 between each incubation period. All sections were quenched with 3% H_2_O_2_/10% for 10 min, followed by several changes of buffer. As a blocking step, sections were then incubated in 7% normal goat serum and 0.1% Triton-X 100 for 2 h at room temperature. This was followed by incubation in primary antibody diluted in 3% normal goat serum and 0.1% Triton-X 100 at 4 °C for 24 h. The antibody used was an anti-TH diluted 1:500 (AB-152, Millipore). After overnight incubation with the primary antibody, sections were rinsed and then incubated for 2 h at room temperature with biotinylated secondary antibodies (anti-rabbit 1:1000; Thermo Scientific) in the same buffer solution. The reaction was visualized with avidin-biotin-peroxidase complex (ABC-Elite, Vector Laboratories), using 3,3-diaminobenzidine as a chromogen. Sections were mounted on super-frost ultra plus slides (Thermo Scientific), dehydrated in ascending alcohol concentrations, cleared in xylene and coverslipped in DPX mounting medium.

For fluorescent immunohistochemistry, free-floating slides were treated with 0.1 M glycine for 5 min in PBS 1× and then with 1% SDS in PBS 1× for 1 min at RT. Slides were blocked with 10% NGS, 1% BSA in PBS 1× for 1 h at RT. The antibodies were diluted in 1% BSA, 0.3% Triton X-100 in PBS 1×. For double immunofluorescence, incubation with primary antibodies was performed overnight at RT and incubation with 1:500 Alexa fluor-conjugated secondary antibodies (Life Technologies) was performed for 2 h at RT. Nuclei were labelled with 1 μg/mL DAPI. Slides were mounted with Vectashield (Vector Laboratories). The following primary antibodies were used: anti-TH 1:1,000 (Sigma-Aldrich or Millipore), anti-FLAG 1:100 (Sigma-Aldrich), anti-MYC 1:100 (Cell Signaling) and anti-Haemoglobin 1:1000 (MP Biomedicals). For detection, Alexa fluor-488 or -594 (Life Technologies) were used. All images were collected using confocal microscopes (LEICA TCS SP2). In the present study, data were collected from 4 independent experiments.

### Quantification of DA neurons in the SNPc

The number of TH-positive cells was determined by counting every fourth 40-µm section as previously described [20]. The delimitation between the ventral tegmental area and the SN was determined by using the medial terminal nucleus of the accessory optic tract as a landmark. All counts were performed blind to the experimental status of the animals through ImageJ software. TH+ cells were counted using the “3D objects counter tool”. Each found object has been quantified applying default settings. The following parameters were modified: Size filter set to 10–20 voxels and the threshold set to 128. Values were expressed as absolute quantification of unilateral SNpc TH+ cells.

### Immunoprecipitation (IP)

Extracts (100 μg) from the substantia nigra (SN) tissue from 3 untreated and 5 mice injected for two months with 1 µL AAV9-CTRL (left SN) or AAV9-Hb (right SN) (titre: 5 × 10^12^ vg/mL) were incubated with 3 µg of the haemoglobin antibody (Cappel, MP Biomedicals, 55039) overnight under constant rotation at 4 °C. Protein G-Sepharose beads (40 μl/tube) were prewashed in the IP buffer (10 mM Tris-Cl pH 7.5, 150 mM NaCl, 2 mM EDTA, 0.5% Triton-X) three times and incubated with the protein/antibody mixtures under constant rotation for 1 h at 4 °C. As a negative control (-), 40 μl/tube of Protein G Sepharose beads was prewashed in the IP buffer three times and incubated with the protein under constant rotation for 1 h at 4 °C, to exclude non-specific binding of α-Syn to IgG and determine the specific interaction between α-Syn and Hb. The precipitant was collected and washed three times with IP buffer to remove non-specifically bound proteins. The beads were resuspended in SDS-PAGE loading buffer (25 μl/tube) and heated at 95 °C for 5 min. After centrifugation of the beads, the supernatant was analyzed by western blotting.

### Statistical analysis

Data represent the mean ± SEM and each group was compared individually with the reference control group using GraphPad Prism (v9) software. To compare the means of two samples, groups were first tested for normality, and then for homogeneity of variance (homoscedasticity). If the normality assumption was not met, data were analyzed by nonparametric Mann-Whitney test. If the normality assumption was met, but the homogeneity of variance was not, data were analyzed by unpaired two-tailed t-test followed by Welch’s correction. If both assumptions were met, data were analyzed by unpaired two-tailed t-test. To compare more than 2 groups one-way ANOVA was used. Statistical analysis of static rods experiments, each group were analyzed by Chi-squared test. Significance to reference samples are shown as *p ≤ 0.05; **p ≤ 0.01; ***p ≤ 0.001; ****p ≤ 0.0001.

### Western blot

iMN9D cells were washed 2 times with PBS 1× and lysed in 300 μL SDS sample buffer 2× (6 well-plate), briefly sonicated, boiled and 10 µl/sample loaded on 15% or 8% (for Spectrin α II immunoblot) SDS-PAGE gel. For antibodies validation, cells were lysed in cold lysis buffer (10 mM Tris-HCl pH 8, 150 mM NaCl, 0.5% Igepal CA-630, 0.5% sodium deoxycholate) supplemented with protease inhibitor mixture (Roche Diagnostics, COEDTAF-RO). Lysates were incubated for 30 min at 4 °C on a rotator and cleared at 12,000 × *g* for 20 min at 4 °C. Supernatants were transferred in new tubes and total protein content was measured using bicinchoninic acid protein (BCA) quantification kit (Pierce) following the manufacturer’s instructions. For SNpc lysates, dissected brain area was lysed in cold RIPA buffer and centrifuged for 10 min at 17,000 × *g*. The sample buffer was added to the supernatant and boiled at 95 °C for 5 min and 30 µg of proteins were loaded on a 10% SDS-PAGE gel. Proteins were transferred to nitrocellulose membrane (Amersham™, Cat. No. GEH10600001) for 1:30 h at 100 V or 16 h 20 V (only for Spectrin α II immunoblot). Membranes were blocked with 5% non-fat milk or 5% BSA (only for Spectrin α II immunoblot) in TBST solution (TBS and 0.1% Tween20) for 40 min at room temperature. Membranes were then incubated with primary antibodies at room temperature for 2 h or overnight at 4 °C (only for Spectrin α II immunoblot). The following antibodies were used: anti-FLAG 1:2000 (Sigma–Aldrich, F3165), anti-MYC 1:2000 (Cell Signaling, 2276), anti-β-actin 1:5000 (Sigma-Aldrich, A5441), anti-Haemoglobin 1:1000 (Cappel, MP Biomedicals, 55039) and anti-GFP 1:1000 (Clontech, 632380), anti-α-syn 1:1000 (C-20) (Santa Cruz Biotechnology, sc-7011-R), anti-α-syn 1:1000 (SYN-1) (BD Transduction Laboratories, 610787), anti-biotin-HRP (Jackson ImmunoResearch Laboratories), anti-Spectrin α II 1:1000 (Santa Cruz, Cat. No. sc-46696), anti-TH 1:1000 (Millipore). For development, membranes were incubated with secondary antibodies conjugated with horseradish peroxidase (Dako) for 1 h at room temperature. Proteins of interest were visualized with the Amersham ECL Detection Reagents (GE Healthcare by SIGMA, Cat. No. RPN2105) or LiteAblot TURBO Extra-Sensitive Chemiluminescent Substrate (EuroClone, Cat. No. EMP012001). Western blotting images were acquired using with Alliance LD2-77WL system (Uvitec, Cambridge) and band intensity was measured UVI-1D software (Uvitec, Cambridge). In the present study, data were collected from 6 independent experiments.

### Production of recombinant human α-syn

Expression and purification of human α-syn were performed as previously described [21]. Briefly, α-syn cDNA was cloned in pET-11a vector and expressed in *Escherichia* *coli* BL21(DE3) strain. Cells were grown in Luria-Bertani medium at 37 °C and expression of α-syn was induced by addition of 0.6 mM isopropyl-β-D-thiogalactoside (IPTG) followed by incubation at 37 °C for 5 h. The protein was extracted and purified according to Huang et al. [22].

### Fibrillation of human α-syn

Lyophilized human α-syn was re-suspended in ddH_2_O, filtered with a 0.22 µm syringe filter and the concentration was determined by absorbance measured at 280 nm. Fibrillization reactions were carried out in a 96-well plate (Perkin Elmer) in the presence of a glass bead (3 mm diameter, Sigma) in a final reaction volume of 200 µL. Human α-syn (1.5 mg/mL) was incubated in the presence of 100 mM NaCl, 20 mM Tris-HCl pH 7.4 and 10 µM thioflavin T (ThT). Plates were sealed and incubated in BMG FLUOstar Omega plate reader at 37 °C with cycles of 50 s shaking (400 r.p.m.) and 10 s rest. The formation of fibrils was monitored by measuring the fluorescence of ThT (excitation: 450 nm, emission: 480 nm) every 15 min. After reaching the plateau phase, the reactions were stopped. Fibrils were collected, centrifuged at 100,000 × *g* for 1 h, resuspended in sterile PBS and stored at −80 °C for further use. For cell culture experiments, the fibrillation reaction was carried out without ThT and PFFs in 0.5 ml conical plastic tubes were sonicated for 5 min in a Branson 2510 Ultrasonic Cleaner prior to addition to the cell culture medium.

### Cleaning procedures

For fibrils inactivation, all contaminated surfaces and laboratory wares, both reusable and disposable, were cleaned using a 1% SDS solution before washing with Milli-Q water according to Bousset et al. [23].

### Atomic force microscopy

Atomic Force Microscopy (AFM) was performed as previously described [24]. Briefly, 3 to 5 μL of fibril solution was deposited onto a freshly cleaved mica surface and left to adhere for 30 min. Samples were then washed with distilled water and blow-dried under a flow of nitrogen. Images were collected at a line scan rate of 0.5–2 Hz in ambient conditions. The AFM free oscillation amplitudes ranged from 25 nm to 40 nm, with characteristic set points ranging from 75% to 90% of these free oscillation amplitudes.

### Cell lines

MN9D-Nurr1^Tet-on^ (iMN9D) cell line stably transfected with pBUD-IRES-eGFP (CTRL cells) or with pBUD-β-globin-MYC IRES-eGFP, 2xFLAG-α-globin (Hb cells) were used [10]. Cells were maintained in culture at 37 °C in a humidified CO_2_ incubator with DMEM/F12 medium (Gibco by Life Technologies, DMEM GlutaMAX® Supplement Cat. No. 31966-021; F-12 Nutrient Mix GlutaMAX® Supplement Cat. No. 31765-027) supplemented with 10% foetal bovine serum (Euroclone, Cat. No. ECS0180L), 100 μg/ml penicillin (Sigma–Aldrich), 100 μg/mL streptomycin (Sigma-Aldrich). 300 μg/ml neomycin (Gibco by Life Technologies, Cat. No 11811-031) and 150 μg/mL Zeocin (Invivogen, Cat. No. ant-zn-05) were used for selection.

### Exposure of iMN9D cells to α-syn monomers and fibrils

iMN9D cells were exposed to 2 μM of α-syn species (2 μM equivalent monomer concentration in the case of amyloids) in cell culture media for 24, 48, and 96 h before collection.

For western blot analysis cells were plated in 6 well-plate (6 × 10^5^ cells/plate for 24 h collection, 3 × 10^5^ cells/plate for 48 h collection, 2 × 10^5^ cells/plate for 96 h collection). Additionally, at 96 h cells were split and maintained for three additional days before collection as Passage 1 (P1). Cells treated with vehicle were used as control (Untreated).

For immunocytochemistry, cells were cultured in 12-well plates with coverslips (3 × 10^5^ cells/well for 24 h collection, 1.5 × 10^5^ cells/well for 48 h collection, 1 × 10^5^ cells/well for 96 h collection).

### Cathepsin D and Calpain inhibitors treatment

Calpain inhibitor III (Santa Cruz Cat. No. SC-201301) and Pepstatin A (Pep, Cathepsin D inhibitor, MedChem Express Cat. No. HY-P0018) were dissolved in dimethylsulfoxide (DMSO) and diluted in cell culture medium to a final concentration respectively of 10 µM and 100 µM. Hb cells were treated with vehicle (DMSO), Calpain inhibitor III and Pepstatin A at the indicated concentrations for 24 h. The medium was then removed and replaced with a new one containing α-syn PFFs, as previously reported, and protease inhibitors to a final concentration respectively of 10 µM and 100 µM, as the day before. Cells were collected at the indicated time points for Western blot analysis. Immunoblot of Spectrin α II was used to monitor Calpain inhibitor activity, while Cathepsin D activity kit was used to monitor Cathepsin D activity. In the present study, data were collected from five independent experiments.

### Cathepsin D activity assay

Cathepsin D (CatD) activity measurements were performed using the Cathepsin D activity assay kit (BioVision, Cat. N. K143) following the manufacturer’s instructions. Briefly, cells were washed twice with PBS, collected in culture media and pelleted by centrifugation at 500 × *g* for 5 min. Cells were counted and 1 × 10 ^5^ cells/well were used. Cells were washed once with PBS, pelleted again by centrifugation at 500 × *g* for 5 min and lysed in CD Cell Lysis Buffer incubating samples for 10 min on ice. Cells were then centrifuged at maximum speed for 10 min. As a control, untreated cells were incubated with PepA (100 µM final concentration) at 37 °C for 10 min prior addition of Reaction Buffer and CD substrate (Positive control). The reaction was left to proceed at 37 °C for 1:30 h in the dark. Fluorescence was read using Thermo Scientific Varioskan® Flash with a 328-nm excitation filter and 460-nm emission filter. CD activity in relative fluorescence units (RFU) was then normalized to Hb cells treated with vehicle and indicated as % Activity. In the present study, data were collected from two independent experiments in duplicate.

### RNA isolation, Reverse Transcription (RT), and quantitative RT-PCR (qRT-PCR)

Total RNA was extracted using TRIzol Reagent (Thermo Fisher, 15596026) and following the manufacturer’s instructions. RNA samples were subjected to TURBO DNase (Invitrogen, Cat. No. AM1907) treatment, to avoid DNA contamination. The final quality of the RNA sample was tested on 1% agarose gel with formaldehyde. A total of 1 μg of RNA was subjected to retrotranscription using iScript™cDNA Synthesis Kit (Bio-Rad, Cat. No. 1708890), according to the manufacturer’s instructions. qRT-PCR was carried out using SYBR green fluorescent dye (iQ SYBR Green Super Mix, Bio-Rad, Cat. No. 1708884) and an iCycler IQ Real-time PCR System (Bio-Rad). The reactions were performed on diluted cDNA (1:2). Mouse actin was used as the normalizing control in all qRT-PCR experiments. The amplified transcripts were quantified using the comparative Ct method and the differences in gene expression were presented as normalized fold expression with ΔΔCt method [25, 26]. The following primer pairs were used:

β-actin: fwd CACACCCGCCACCAGTTC, rev CCCATTCCCACCATCACACC;

Capn1: fwd TTGACCTGGACAAGTCTGGC, rev CCGAGTAGCGGGTGATTATG;

Capn2: fwd ATGCGGAAAGCACTGGAAG, rev GACCAAACACCGCACAAAAT;

Ctsd: fwd CAGGACACTGTATCGGTTCCA, rev CAAAGACCGGAAGCACGTTG.

## Results

### Long-term Hb overexpression in SNpc triggers loss of DA neurons, decreases motor performances and causes cognitive impairments

**Fig. 1:**
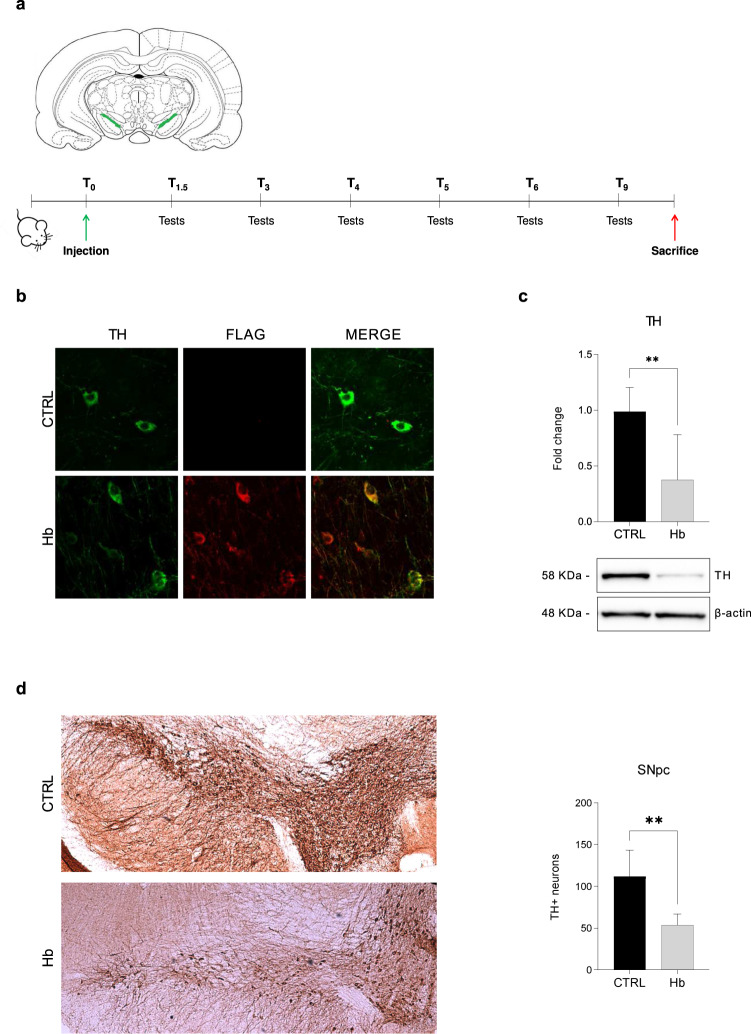
Hb overexpression in SNpc triggers the loss of DA neurons. Scheme representing the experimental protocol used for the assessment of Hb overexpression. AAV9 expressing Hb (Hb) and CTRL (CTRL) constructs were bilaterally injected in the brain of 3-month-old mice. Brain diagram indicating SNpc (green) as the region of the injection (upper panel). Behavioural tests were performed to verify the locomotor performance of mice 1.5, 3, 4, 5, 6 and 9 months post-injection (lower panel) (**a**). Co-localization of FLAG-Hb in DA TH + neurons (**b**). The level of expression in SNpc of Hb and CTRL mice (*n* = 4) at 10 months post-injection of tyrosine hydroxylase (TH, 58 KDa) was assessed by Western blot (**c** lower panel). Band intensity was quantified (**c** upper panel). TH-positive neurons of the SNpc were evaluated by immunohistochemistry (**d** left panel) and quantified (**d** right panel) for Hb and CTRL mice (*n* = 4; 3 slices each). Data represent means ± SEM. Statistical analysis was performed with unpaired t-test with Welch’s correction. *p ≤ 0.05; **p ≤ 0.01; ***p ≤ 0.001; ****p ≤ 0.0001.

To study the effects of Hb overexpression on endogenous α-syn in vivo, we injected a mixture of AAV9-2xFLAG-α-globin and AAV9-β-globin-MYC (indicated as AAV9-Hb) or AAV9-CTRL bilaterally into the SNpc of 3 months old mice (respectively named as Hb and CTRL mice). In a previous study [11], over-expression of Hb for one month in SNpc of mice infected with the very same AAV9-Hb provoked the formation of cytoplasmic and nucleolar Hb aggregates and a mild motor learning impairment but did not impact DA cells viability. To investigate long-term Hb effects on DA neurons’ homoeostasis and mice behaviour, here we prolonged Hb overexpression up to 9 months after the injection (Fig. 1a).

We first proved by immunofluorescence that ectopic expression of Hb was maintained in DA neurons in the timeframe of the experiment (Fig. 1b). Interestingly, Hb mice presented a decrease of tyrosine hydroxylase (TH) expression of about 50% by Western blot analysis (Fig. 1c). To understand whether the decrease in TH levels was due to a loss of neurons or a change in expression levels, we performed immunohistochemistry analysis of brain tissues from Hb and CTRL mice and quantified DA cells in SNpc. A significant loss of about 50% of DA neurons was evident in SNpc of Hb mice, indicating that long-term overexpression of Hb was detrimental to DA neurons (Fig. 1d).

**Fig. 2:**
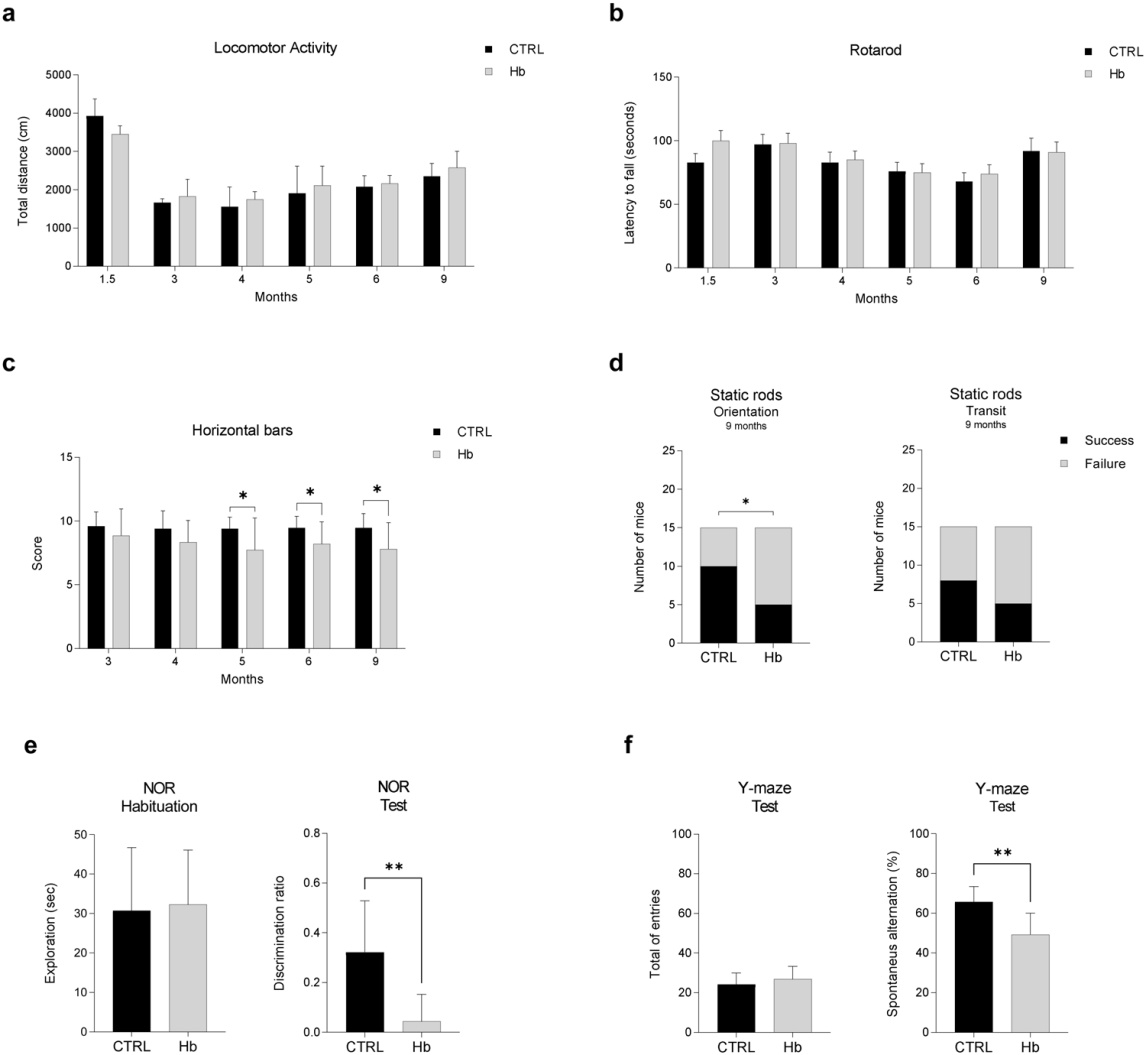
Hb overexpression in SNpc decrease motor performances and trigger cognitive impairments. CTRL (*n* = 12) and Hb (*n* = 12) mice were assessed for locomotor activity and total distance (cm) was recorded at different time points after injections (**a**). CTRL (*n* = 15) and Hb (*n* = 15) mice were also scored for motor coordination with the rotarod test (**b**) and latency to fall was measured. Horizontal bars test was used to assess forelimb strength and coordination and CTRL (*n* = 15) and Hb (*n* = 15) mice were scored for their performance(**c**). Data represent means ± SEM. Statistical analysis was performed with unpaired t-test with Welch’s correction. *p ≤ 0.05. CTRL (*n* = 15) and Hb (*n* = 15) were assessed in static rods test measuring two parameters, transit time and orientation time (seconds) and different time points. In panel (**d**) it is depicted the results at 9 months after injection. Chi-square test was used to evaluate the success/failure of each group. *p ≤ 0.05. Novel object recognition test (NOR) was used to evaluate recognition memory (**e**). CTRL (*n* = 8) and Hb (*n* = 8) was habituated to the object for 10 min and exploration (seconds) of the objects was measured (**e** left panel). After 1 h, mice were assessed to recognize the novel object and the discrimination ratio was plotted (**e** right panel). Spontaneous alternation in the Y-maze was used to measure spatial working memory in CTRL (n = 10) and Hb (*n* = 12) mice (**f**). Total entries were calculated for each group (**f** left panel). Spontaneous alternation % was plotted for each group (**f** right panel). Data represent means ± SEM. Statistical analysis was performed with unpaired t-test with Welch’s correction. *p ≤ 0.05; **p ≤ 0.01.

To determine the behavioural impact of Hb overexpression, we monitored Hb and CTRL mice by a series of tests over a period of 9 months after the injection (Fig. 1a). No major difference in locomotor activity and rotarod performance was observed between the two groups at all the time points examined (Fig. 2a, b). However, some deficits in Hb mice were evident by applying more specific tests. In horizontal bars, a test that measures the forelimb strength and coordination, Hb mice performed worse than CTRL animals starting from 5 months after the injection (Fig. 2c). In static rods, a test to evaluate the coordination of mice to walk on wooden rods of different diameters, Hb mice performed worse on narrow rods (10 mm). Hb mice often fall out of the rods both during orientation and transit, and they failed to complete the test significantly more often than CTRL mice, in particular those at the 9 months post-injection time point (Fig. 1d; Supplementary Fig. 1).

Since PD patients may experience cognitive dysfunctions [1], we tested mice in two cognitive tests, the novel object recognition test (NOR) and the Y-maze for spontaneous alternation, assessing recognition memory and spatial working memory, respectively. In the NOR, Hb mice showed a strong deficit in recognizing the novel object, as indicated by the discrimination ratio (Fig. 2e). In the Y-maze, Hb mice displayed less spontaneous alternation compared to CTRL mice with no difference in the total entries in the arms (Fig. 2f).

These data demonstrate that Hb expression in SNpc caused partial loss of DA neurons and induced mild motor impairments and significant cognitive deficits.

### Biochemical analysis of endogenous α-syn upon Hb overexpression in vivo

**Fig. 3:**
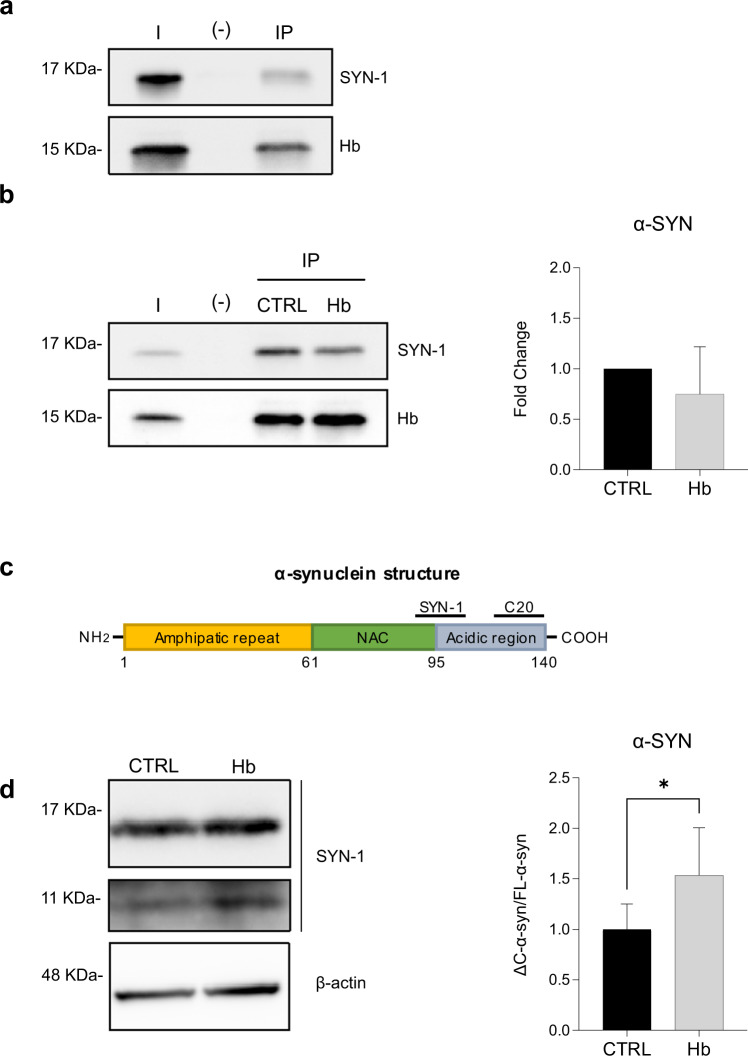
In vivo investigation of the interplay between α-syn and Hb. Representative immunoblotting with SYN-1 (**a** upper membrane) and Hb (**a** lower membrane) antibodies of the SN tissue of WT mice (*n* = 3) subjected to Hb immunoprecipitation. Representative immunoblotting with SYN-1 (**b** upper membrane) and Hb (**b** lower membrane) antibodies of the SN tissue of CTRL and Hb mice (*n* = 5) subjected to Hb immunoprecipitation. Hb staining (**a** and **b**) has been used as internal control. Schematic representation of the domain structure of human α-syn protein and epitopes of anti-α-syn antibodies used for the study (**c**). FL-α-syn (17 KDa), ΔC-α-syn (11 KDa) expression in SN of Hb and CTRL mice (n = 8) at 10 months post-injection of (**d** left panel) was assessed by western blot. Band intensity was quantified (**d** right panel). I, input; (-), control resin with no antibody; IP, immunoprecipitated samples using anti-Hb antibody (see “Materials and methods”). Data represent means ± SEM. Statistical analysis was performed with unpaired *t*-test with Welch’s correction. *p ≤ 0.05.

Given the reported presence of Hb-α-syn complexes in primate brains and red blood cells [15, 16], we investigated whether α-syn and Hb interact. We first evaluated the interaction of endogenous α-syn and Hb in the SN of WT mice. As shown in Fig. 3a, the presence of α-syn was detected in Hb immunoprecipitate. The very same co-immunoprecipitation experiment was carried out from the SN of AAV9-Hb and AAV9-CTRL injected mice confirming their interaction. Upon Hb overexpression, α-syn levels seemed to be lower although this difference did not reach statistical significance (Fig. 3b).

We then studied the post-translational processing of endogenous α-syn by western blot analysis with two epitope-specific antibodies: C-20 and SYN-1, recognizing the full-length (FL-α-syn) or the C-terminal truncated (ΔC-α-syn) isoforms, respectively (Fig. 3c). Interestingly, a statistically significant increase in ΔC-α-syn/FL-α-syn ratio was observed in the SNpc of mice overexpressing Hb compared to the control group (Fig. 3d). C-terminal cleavage is one of the major post-translational modifications (PTM) of α-syn [26]. It could be particularly detrimental as ΔC-α-syn self-assembles into fibrils and increases the aggregation rate and toxicity in both cultured cells [27, 28] and animal models [29–31].

In the remaining part of the study, we investigated the potential role of Hb in the formation of α-syn C-terminal fragments.

### Biochemical analysis and structural characterization of α-syn preformed fibrils (PFFs) preparation

Exogenous administration of α-syn PFFs to cell lines expressing endogenous α-syn leads to the templated misfolding and aggregation of the endogenous protein into Lewy Body-like structures that contain α-syn C-terminal fragments [8, 9]. We, therefore, investigated whether Hb overexpression in vitro increased α-syn truncation. To this end, we produced and characterized α-syn PFFs. Fibrillation reactions were monitored by thioflavin T (ThT) fluorescence and PFFs were collected at plateau as long fibrils (Supplementary Fig. 2a). Atomic force microscopy (AFM) and Western Blot were performed as previously reported [32] to characterize the structure and molecular weight distribution of the PFFs preparation (Supplementary Fig. 2b). Both Monomers (Ms) and PFFs preparations contained monomeric and dimeric α-syn. (Supplementary Fig. 2c).

As a cellular model system, we took advantage of a DA iMN9D mouse cell line stably overexpressing α and β-globins and forming α_2_β_2_ tetramer, as previously described [11, 12] (from hereafter named “Hb cells”). We included iMN9D cells stably transfected with a mock vector as a control line [11, 12, 33].

**Fig. 4:**
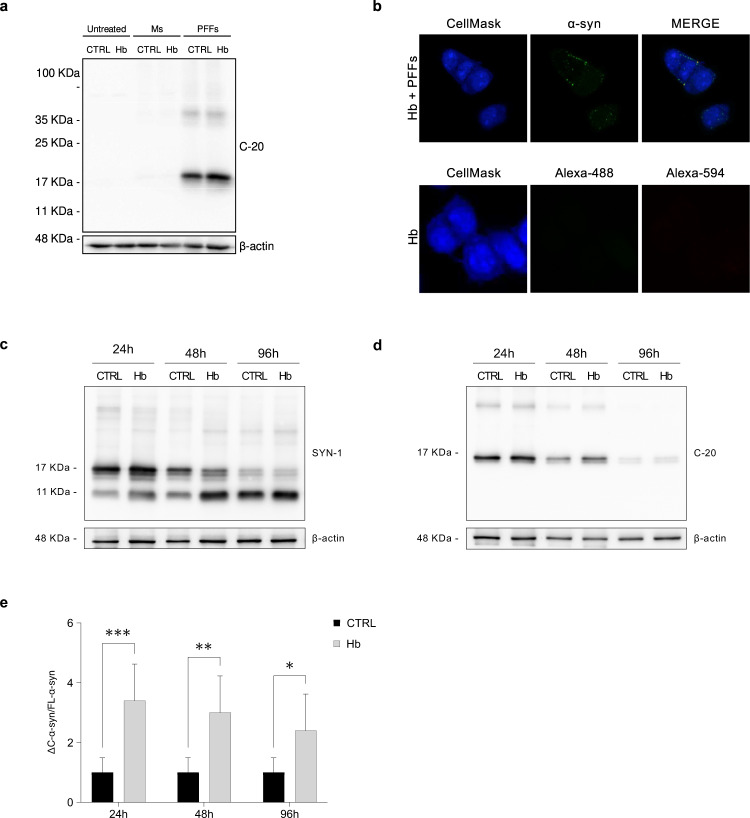
Impact of Hb overexpression on the accumulation of C-terminal truncated α-syn. Representative Western blot of lysates from Hb and CTRL cells upon supplementation of vehicle, Ms and PFFs for 24 h (**a**). Representative confocal microscopy images of Hb cells supplemented with Alexa-488 labelled PFFs for 24 h. Entire cells were labelled by CellMask. Untransfected cells incubated only with secondary antibody were used to establish autofluorescence levels. Nuclei were stained with DAPI. Scale bar 10 μm (**b**). CTRL and Hb cells were treated with α-syn amyloids. Cell lysates were collected at the indicated time points and analyzed by immunoblotting with SYN-1 (**c)** and C-20 antibodies (**d**). Band intensity corresponding to ΔC-α-syn and FL-α-syn was quantified and the ratio was calculated. Data represent means ± SEM and are representative of six independent experiments. Statistical analysis was performed with one-way Anova. *p ≤ 0.05; **p ≤ 0.01; ***p ≤ 0.001 (**e**).

2 μM of Alexa-488 labelled PFFs and Ms were added to the culture medium of Hb and control cells for 24 h. The intracellular accumulation of PFFs, but not of Ms, was confirmed by Western blot in both cell lines (Fig. 4a) as well as by immunofluorescence. Intracellular fluorescent punctate structures were evident in PFFs-treated cells, demonstrating PFFs internalization in both cell types (Fig. 4b). To exclude that the Alexa488-labelled PFFs could be just attached to the outer membrane and not internalized, we incubate PFFs-treated Hb and control cells with Trypan Blue before fixation. Trypan blue is a viable-impermeable dye that quenches green fluorescence, and therefore selectively quenches non-internalized Alexa488-labelled PFFs (data not shown) [19].

### Hb triggers the accumulation of a C-terminal truncated form of α-syn in vitro

Hb and control cells were then treated with 2 μM PFFs for 24, 48, and 96 h and the pattern of α-syn species was studied by Western blot analysis using SYN-1 or C-20 antibodies. In general, the intracellular expression of FL-α-syn decreased over time, whereas ΔC-α-syn species increased (Fig. 4c, d). Notably, ΔC-α-syn was reproducibly more abundant in Hb than CTRL cells at each time point and the levels of ΔC-α-syn normalized to FL-α-syn (ΔC-α-syn/FL-α-syn ratio) were significantly higher in Hb than CTRL cells along with the timeframe of the experiment (Fig. 4c, e). These results recapitulate our in vivo observation of an increase of truncated α-syn species upon overexpression of Hb, therefore corroborating our findings.

### Proteases contribution to the accumulation of α-syn C-terminal truncated species

To identify the endogenous protease/s that cleave/s α-syn in our experimental settings, RNA-seq data of Hb cells were interrogated [19] and showed that these cells mainly express Calpain I (Capn I) and Cathepsin D (Ctsd), making them promising candidates for α-syn cleavage.

To investigate the role of Capn I, we treated Hb cells with the specific inhibitor Capn inhibitor III (CI-III) and measured ΔC-α-syn/FL-α-syn ratio. Hb cells were pre-treated with either 10 µM CI-III or the vehicle (DMSO) for 24 h, then incubated with PFFs for 24 or 48 h.

**Fig. 5:**
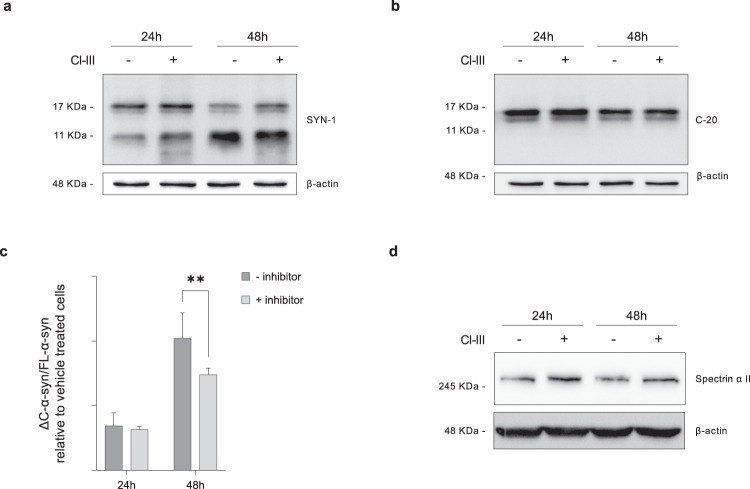
Effect of Calpain I inhibition on α-syn C-terminal truncated species accumulation in Hb cells. Cell lysates of Hb cells treated with DMSO (-) and Calpain inhibitor III (+) were analyzed by immunoblotting with SYN-1 (**a**) and C-20 (**b**) antibodies. Band intensity corresponding to ΔC-α-syn and FL-α-syn was quantified and the ratio was calculated. Data represent means ± SEM and are representative of six independent experiments. Statistical analysis was performed with one-way Anova. *p ≤ 0.05; **p ≤ 0.01 (**c**). Cell lysates of Hb cells treated with DMSO (-) and CI-III (+) were analyzed by immunoblotting with anti-Spectrin α II antibody (**d**).

Western Blot analysis of cell lysates revealed that ΔC-α-syn/FL-α-syn ratio decreased in a statistically significant manner upon 48 h of CI-III treatment, demonstrating that Capn I is involved in α-syn C-terminal truncation in our experimental settings (Fig. 5a–c). Cells treated with CI-III also showed increased levels of α-spectrin, a well-known Capn I substrate, therefore confirming successful calpain I inhibition (Fig. 5d).

The very same experimental settings were then used to assess the role of Ctsd. As shown in Supplementary Figure 3, 100 µM of Pepstatin A, an inhibitor of acid proteases including Ctsd, did not modify the pattern of α-syn truncation. It should be considered that in our experimental conditions Pepstatin A was able to inhibit only 20 or 30% of Ctsd activity, respectively at 24 and 48 h upon PFFs supplementation (Supplementary Fig. 3; see “Material and methods”). Given that higher Pepstatin A concentration led to cell death, additional experiments are needed to definitively rule out Ctsd involvement in Hb-induced α-syn fragments formation. These results show that the formation of α-syn fragments induced by Hb depends, at least in part, by Calpain activity, a protease previously shown to be involved in α-syn PTM.

## Discussion

Hb is attracting interest in the field of PD for some intriguing observations: i. Hb expression in the mesencephalic DA cell system correlates to cell’s vulnerability in disease [11]; ii. Hb is localized, but not exclusively, in the mitochondria, an organelle crucial for PD pathogenesis, and it influences its activity [14]; iii. its association to mitochondria decreases in aging and post-mortem PD brains [15–17]; iv. Hb overexpression increases DA cells’ susceptibility to neurochemical intoxication, a PD cellular model [11]; v. Hb inhibits stress-induced autophagy, a pathway involved in PD [11].

Here, we show that long-term overexpression of Hb in mouse SN leads to a loss of 50% of DA neurons after 9 months from the injection. We previously showed that after one month, Hb was forming intracellular aggregates localized in the cytoplasm and to the nucleolus leading to initial impairment of motor learning with no cellular loss [11]. Here, we monitored AAV-injected mice for behavioural deficits for all 9 months, since the onset of behavioural alterations was expected to be slow and progressive. Hb mice presented subtle motor deficits and evident cognitive dysfunctions. Locomotor activity and rotarod test did not evidence overt motor impairments. However, two tests that evaluate different motor skills showed a decrease in motor abilities of Hb mice. These results are in agreement with what is observed in PD patients and animal models, where motor symptoms appear when most of the dopaminergic neurons are lost [1]. Hb mice showed substantial defects in memory assessment and spatial working memory. This is consistent with the fact that cognitive impairments usually appear in PD patients before the onset of motor symptoms [34]. Similar deficits have been observed in a PD mouse model with bilateral partial 6-hydroxydopamine lesions and with a loss of SNpc DA neurons of about 60%. These mice showed a mild motor phenotype (e.g. no locomotor activity alteration) and cognitive deficits, as evidenced in NOR test and other behavioural assays not related to motor functions [35]. It is therefore intriguing that the overexpression of Hb is phenocopying features of a well-accepted neurochemical model of PD.

It remains unclear how Hb is triggering DA cells’ loss. To answer this question, here we provide evidence that Hb overexpression in vivo and in vitro triggers ΔC-α-syn formation. Pathologic α-syn presents several PTMs and C-terminal truncations represent a prominent feature of the protein in the inclusion bodies [25, 36]. ΔC-α-syn species are known to increase the pathological aggregation into LB inclusions because of their propensity to aggregate, to promote the aggregation of FL-α-syn and to trigger toxicity. Such a prion-like seeding mechanism of ΔC-α-syn has been observed both in vitro and in vivo [25, 26, 37, 38].

In post-mortem PD brains, LB inclusions in SNpc neurons comprise a 20% of amyloidogenic ΔC-α-syn [1, [26, 39]. Since α-syn fragments accumulate in the vermiform appendix, the fact that patients with appendectomy are less likely to develop PD or present a delayed disease onset is considered in support of the notion that α-syn carboxy-truncations might be relevant in disease pathogenesis [40]. The control of the proteolytic cleavage of α-syn is therefore a crucial event and a viable target for therapeutics.

Endogenous ΔC-α-syn content in SNpc of Hb mice showed an increase of about 50% compared to CTRL mice. These results were recapitulated in vitro where iMN9D cells overexpressing α and β chains of Hb were challenged with PFFs [4, 7]. PFFs were internalized by Hb iMN9D cells and promoted the formation of α-syn fragments. ΔC-α-syn/FL-α-syn ratio increased significantly in iMN9D Hb cells as compared to control and provided further evidence that Hb expression correlates with the C-terminal truncation of α-syn. The repertoire of candidate proteases involved in the formation of ΔC-α-syn comprises neurosin, Capn I, Ctsd and matrix metalloproteinase-3 [37]. Our data show that Hb leads to activation of calpain I, since ΔC-α-syn is significantly inhibited by CI-III. Interestingly, calpain inhibitors were shown to exert disease-modifying activity and reduction of α-syn deposition in transgenic models of PD [41]. Additional experiments should address the involvement of Ctsd since its inhibitor, Pepstatin A, is highly toxic in our experimental setting. It remains unclear how Hb is linked to calpain activation. Given the observation that the Hb-α-syn complex can accumulate in the cytoplasm of striatal and SN cells in monkeys, in human post-mortem brains and red blood cells [15, 16], we investigated whether Hb and α-syn were forming complexes in our experimental conditions. Interestingly, we confirmed these observations in mice, showing that endogenous α-syn and Hb interact in WT animals and that these results are recapitulated upon Hb overexpression in vivo. Additional studies are needed to understand the role of this interaction in α-syn cleavage. Importantly, the formation of endogenous ΔC-α-syn fragments could be a crucial step in the chain of events from Hb expression to DA cell loss in vivo. In support of this model, loss of DA neurons has been observed in mice overexpressing ΔC-α-syn [42, 43]. They also showed deficits in locomotion and cortical-hippocampal memory tests [29]. Moreover, passive immunization against ΔC-α-syn ameliorated neurodegeneration and neuroinflammation, reduced the accumulation of ΔC-α-syn and improved motor and memory deficits in a mouse model of PD [44]. It will be interesting to investigate whether the amount of endogenous ΔC-α-syn in Hb mice is sufficient to promote neurodegeneration or it requires a second pathological event triggered by Hb.

Our results may be of interest beyond PD. The presence of inclusion bodies rich in fibrillar FL-α-syn and ΔC-α-syn fragments is a common characteristic of synucleinopathies, a group of neurodegenerative diseases that include Lewy Body Dementia (LBD) and Multiple System Atrophy (MSA). In MSA, the typical α-syn-containing glial cytoplasmic inclusions are restricted to oligodendrocytes [45]. Interestingly, Hb is expressed in mouse and human oligodendrocytes in vitro and in vivo [10], and MSA mouse models and post-mortem brains show an increase of Hb expression of 2.5- to 3-fold [46, 47].

These results suggest the need for further studies on the role of Hb in these neurodegenerative diseases.

## Conclusion

Our study provides new evidence for an important role of Hb in PD pathogenesis. Given the effects of Hb overexpression on DA cells’ viability and α-syn aggregation propensity, an analysis of the correlation between genetic variation of Hb genes and Hb levels in the brain is needed to potentially associate its expression to the onset of synucleinopathies, including PD.

## Supplementary information

Supplementary Figure Legends

Supplementary Figure 1

Supplementary Figure 2

Supplementary Figure 3

Original Data File

Checklist

## Data Availability

All data generated during this study have been included in the manuscript. No data are deposited in databases.
