# Supplementary material for: Neuronal haemoglobin induces loss of dopaminergic neurons in mouse Substantia nigra, cognitive deficits and cleavage of endogenous α-synuclein
Source: Cell Death Dis. 2022 Dec 16;13(12):1048. doi: 10.1038/s41419-022-05489-y (PMC9758156; doi:10.1038/s41419-022-05489-y)
Supplement: Supplementary file 1 — Supplementary Figure Legends [file 41419_2022_5489_MOESM1_ESM.docx]

**SUPPLEMENTARY INFORMATION**

**Additional file 1:**

**Supplementary Figure 1. Static rods test on CTRL and Hb mice.** CTRL (n=15) and Hb (n=15) mice were assessed in static rods test measuring two parameters, transit time and orientation time (seconds) and different time points. Different diameters of the rods were used, 35, 25 and 15mm. The time points evaluated in this test were 4 (**a**), 5 (**b**), 6 (**c**) and 9 months (**d**) after injection. Data represent means ± SEM. Statistical analysis was performed with unpaired t-test with Welch’s correction. *, p ≤ 0.05.

**Additional file 2:**

**Supplementary Figure 2. Biochemical analysis and structural characterization of α-syn preparations.** Fibrillation curve of recombinant human α-syn protein analysed using ThT fluorescence assay. The mean of three wells is represented (**a**). Representative 5 μm x 5 μm AFM micrograph of fibrillary human α-syn aggregates after 5 min of sonication (**b**). Ms and PFFs preparations were analysed by Western blot with SYN-1 and C20 antibodies. The same membrane was exposed for a longer period to detect high molecular weight species (**c**).

**Additional file 3:**

**Supplementary Figure 3. Effect of Ctsd inhibition on α-syn C-terminal truncated species accumulation in Hb cells.** Cell lysates of Hb cells treated with DMSO (-) and Pepstatin A (+) were analysed by immunoblotting with SYN-1 (**a**) and C-20 (**b**) antibodies. Band intensities corresponding to ΔC-α-syn and FL-α-syn were quantified and the ratio was calculated. Data represent means ± SEM and are representative of five independent experiments (**c**). Cell lysates of Hb cells treated with DMSO (-) and Pepstatin A (+) were analysed by Ctsd activity assay. Data represent means ± SEM and are representative of two independent experiments, each performed in three replicas and are expressed as a percentage of vehicle-treated cells (**d**). Statistical analysis was performed with one-way Anova. *, p ≤ 0.05; **, p ≤ 0.01; ^***^, p≤ 0.001; ****, p ≤ 0.0001.
