# Supplementary figures and images for: Neuronal haemoglobin induces loss of dopaminergic neurons in mouse Substantia nigra, cognitive deficits and cleavage of endogenous α-synuclein
Source: Cell Death Dis. 2022 Dec 16;13(12):1048. doi: 10.1038/s41419-022-05489-y (PMC9758156; doi:10.1038/s41419-022-05489-y)

Supplementary Figure 1

a

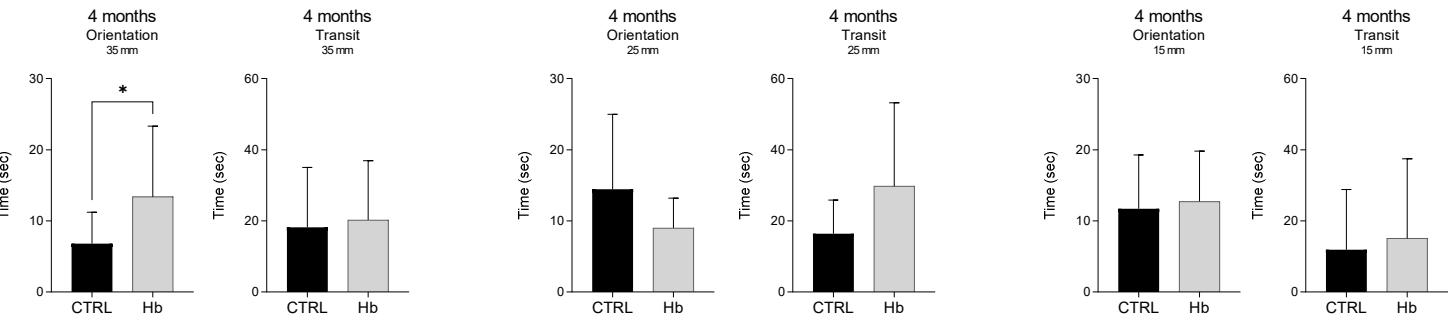

b

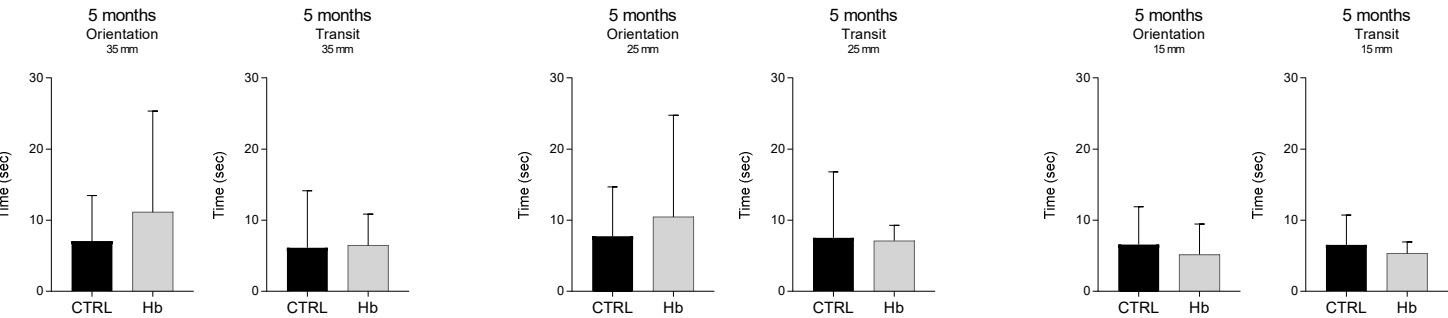

c

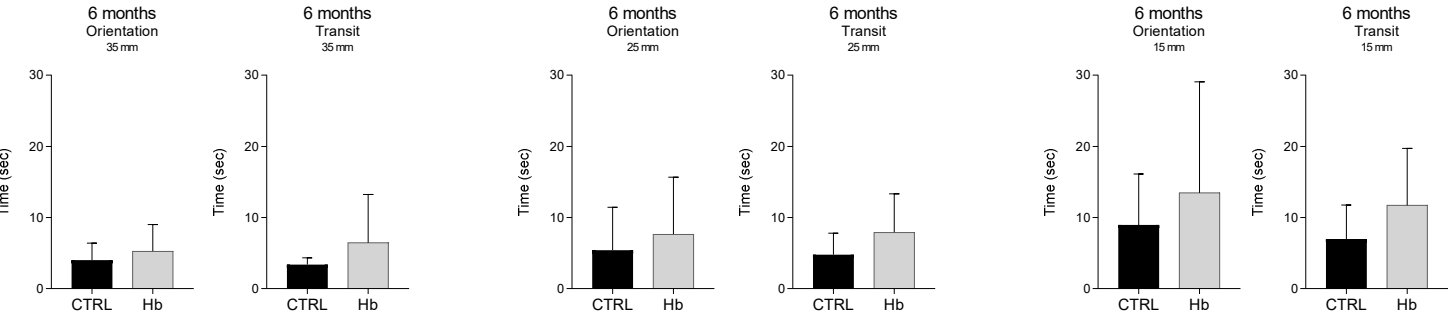

d

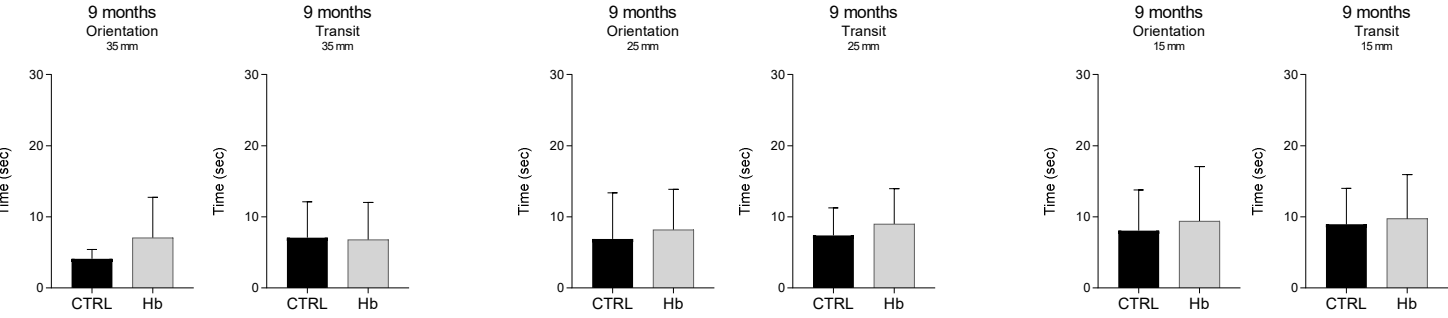

Supplement: Supplementary file 2 — Supplementary Figure 1 [file 41419_2022_5489_MOESM2_ESM.pdf]

Supplementary Figure 2

a

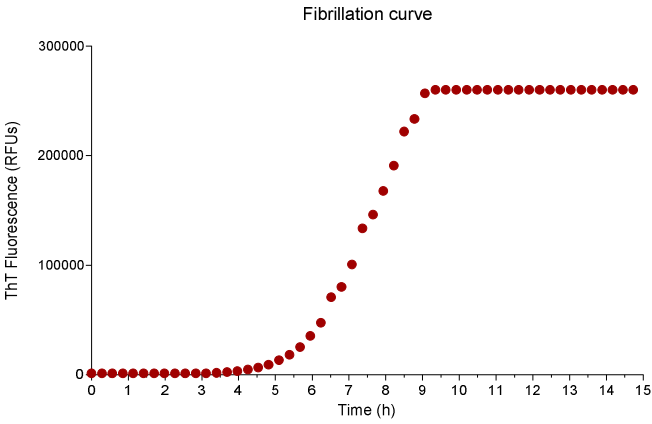

b

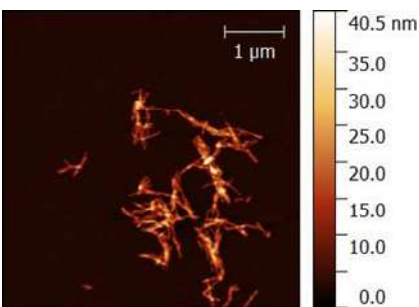

c

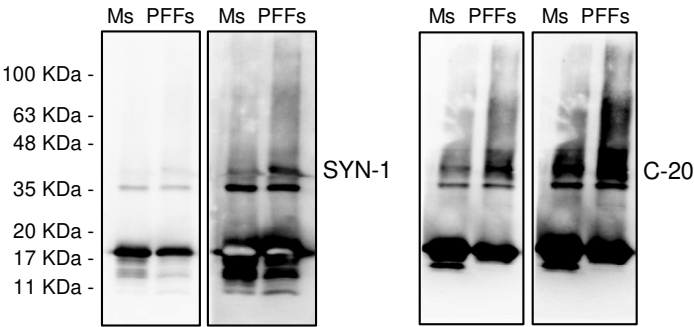

Supplement: Supplementary file 3 — Supplementary Figure 2 [file 41419_2022_5489_MOESM3_ESM.pdf]

Supplementary Figure 3

a

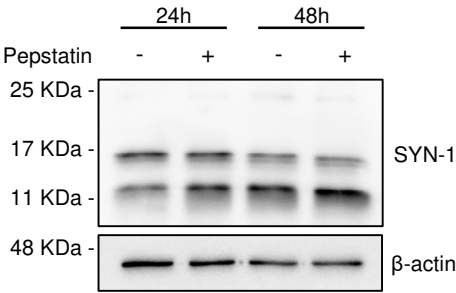

b

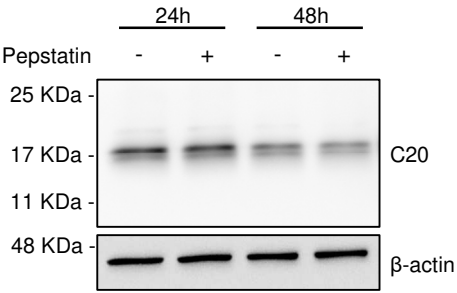

c

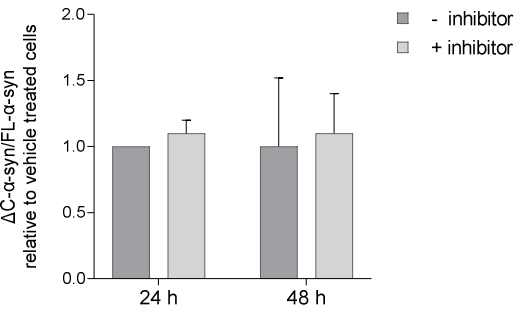

d

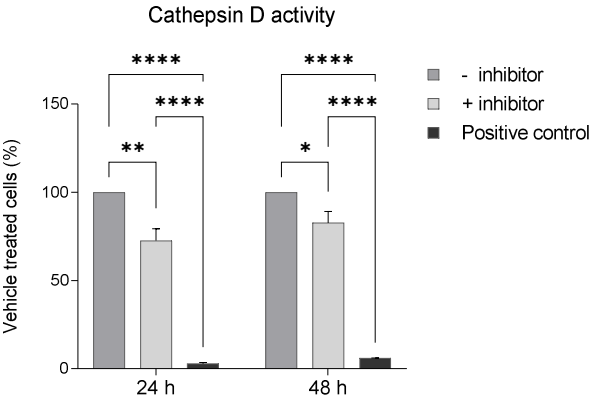

Supplement: Supplementary file 4 — Supplementary Figure 3 [file 41419_2022_5489_MOESM4_ESM.pdf]
