# Supplementary material for: Neuronal haemoglobin induces loss of dopaminergic neurons in mouse Substantia nigra, cognitive deficits and cleavage of endogenous α-synuclein
Source: Cell Death Dis. 2022 Dec 16;13(12):1048. doi: 10.1038/s41419-022-05489-y (PMC9758156; doi:10.1038/s41419-022-05489-y)

Figure 1

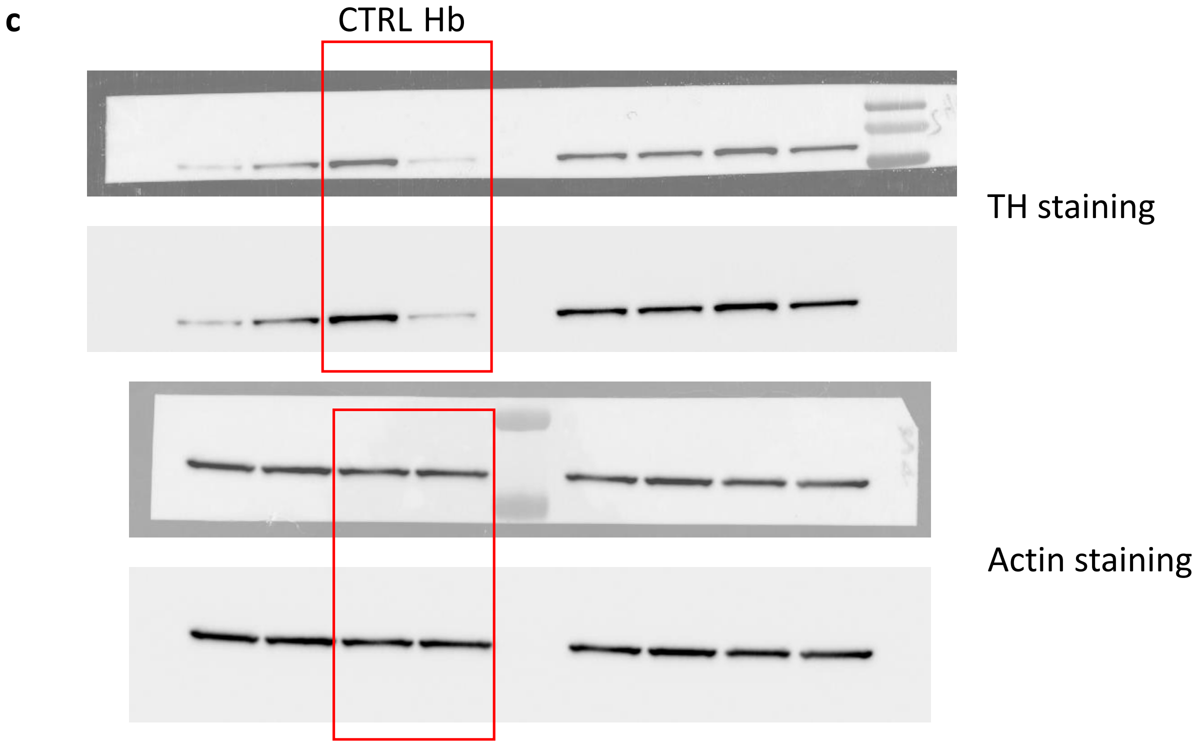

Figure 3

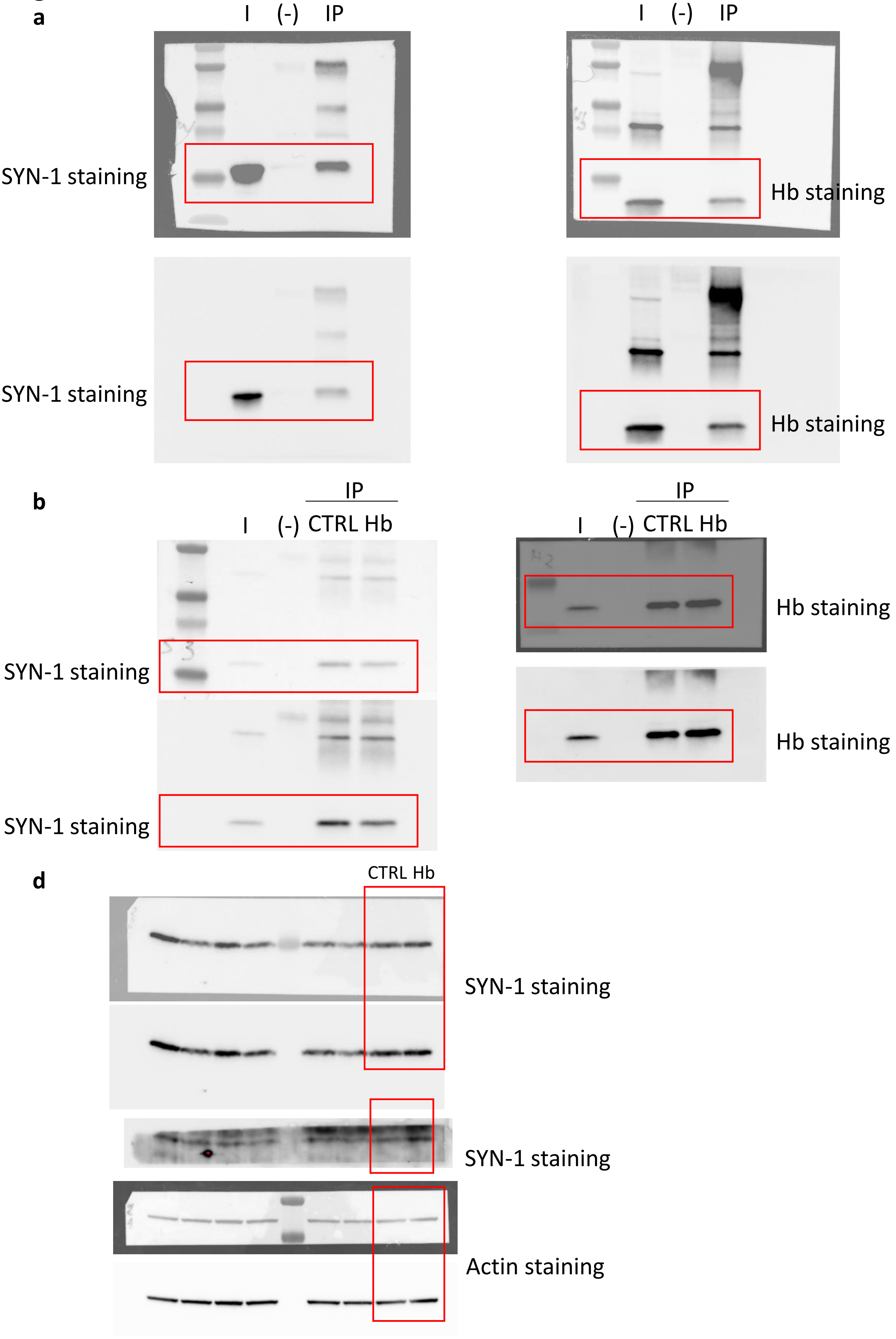
a
I (-) IP
SYN-1 staining
SYN-1 staining
I (-) IP
Hb staining
Hb staining
b
IP
I (-) CTRL Hb
SYN-1 staining
SYN-1 staining
IP
I (-) CTRL Hb
Hb staining
Hb staining
d
CTRL Hb
SYN-1 staining
SYN-1 staining
Actin staining

Figure 4

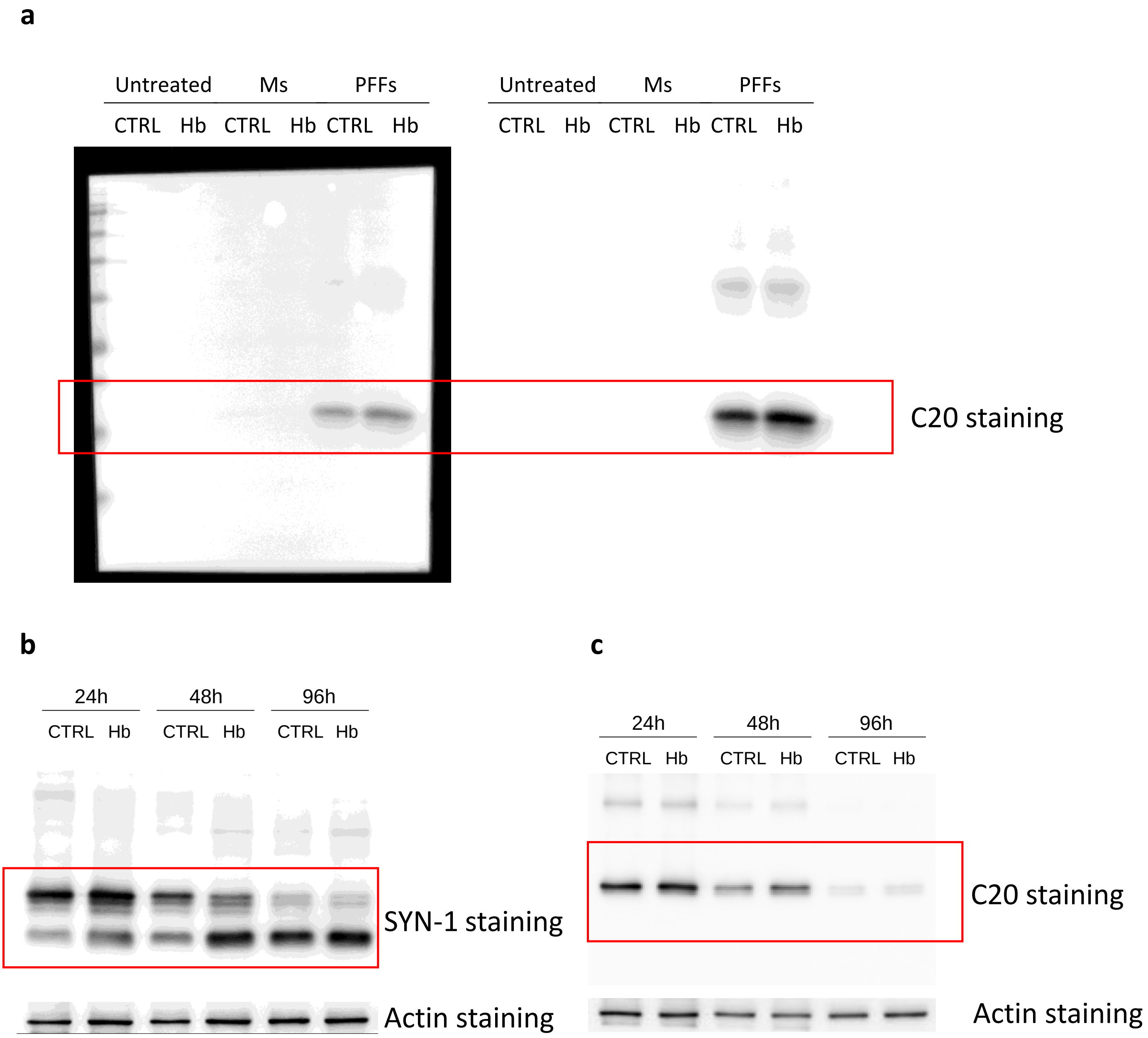
a
Untreated
Ms
PFFs
CTRL
Hb
CTRL
Hb
CTRL
Hb
Untreated
Ms
PFFs
CTRL
Hb
CTRL
Hb
CTRL
Hb
C20 staining
b
24h
48h
96h
CTRL
Hb
CTRL
Hb
CTRL
Hb
SYN-1 staining
Actin staining
c
24h
48h
96h
CTRL
Hb
CTRL
Hb
CTRL
Hb
C20 staining
Actin staining

Figure 5

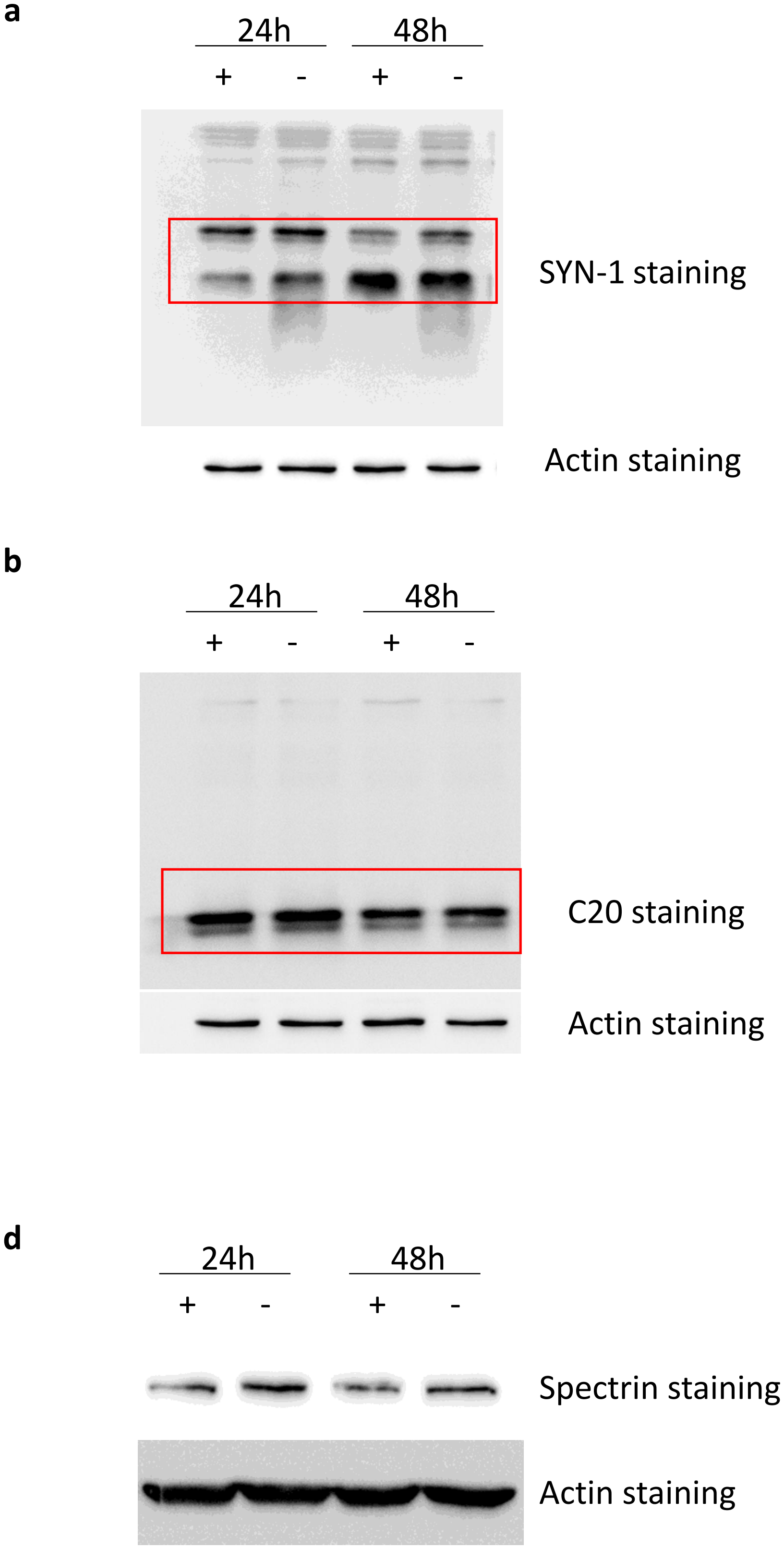
a
24h
48h
+
-
+
-
SYN-1 staining
Actin staining
b
24h
48h
+
-
+
-
C20 staining
Actin staining
d
24h
48h
+
-
+
-
Spectrin staining
Actin staining

Supplementary Figure 2

c

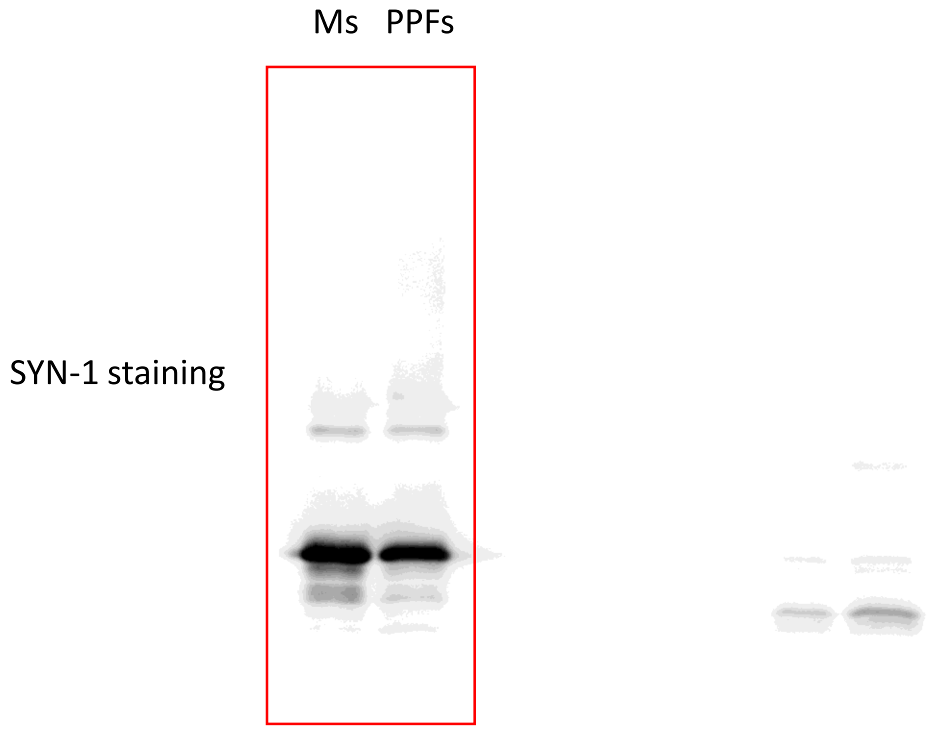

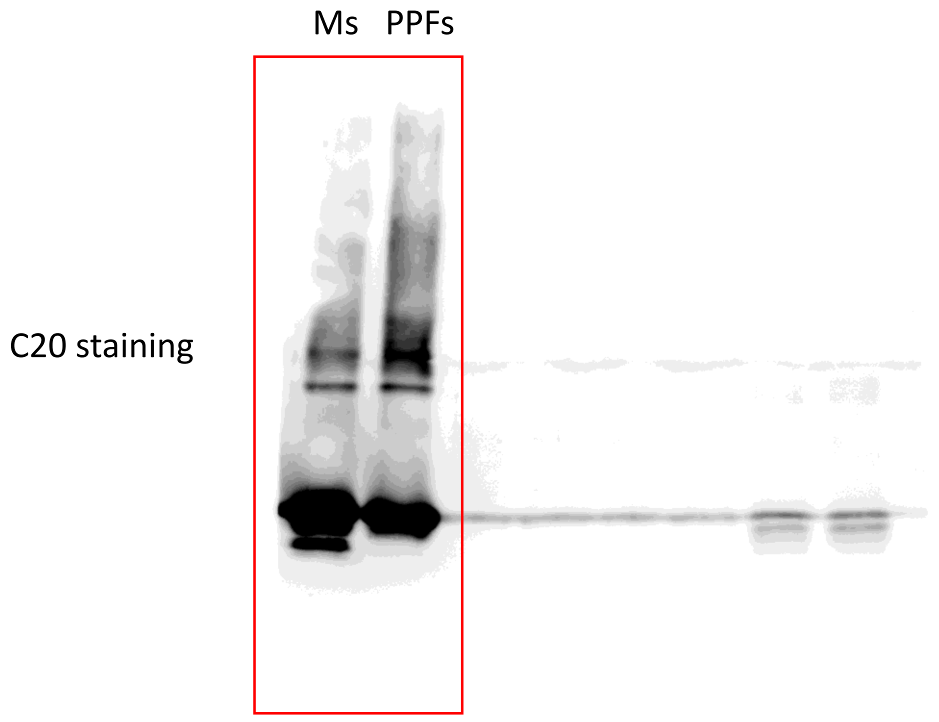

Supplementary Figure 3

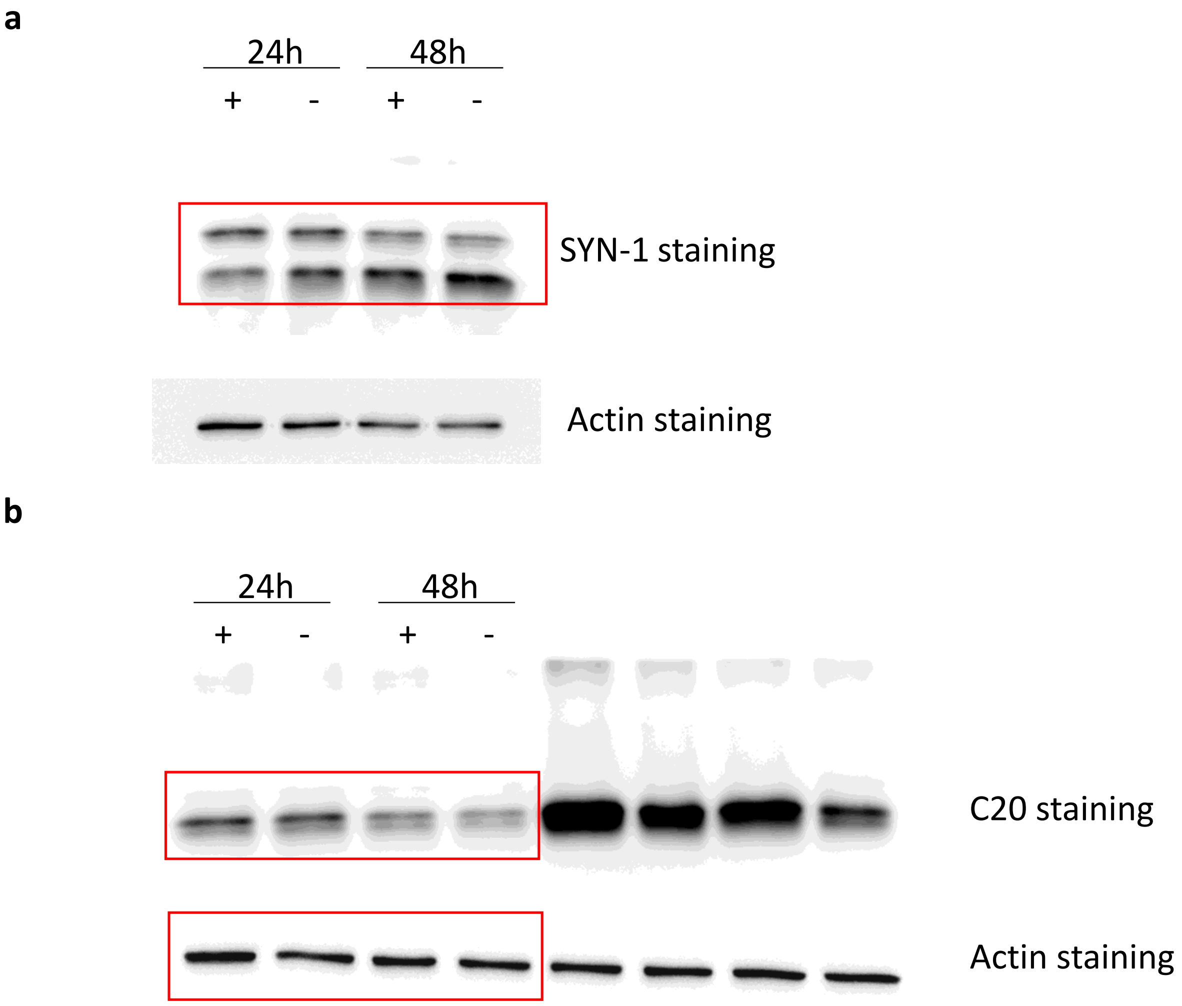

Supplement: Supplementary file 5 — Original Data File [file 41419_2022_5489_MOESM5_ESM.pdf]
